# Exploring lithium’s transcriptional mechanisms of action in bipolar disorder: a multi-step study

**DOI:** 10.1038/s41386-019-0556-8

**Published:** 2019-10-25

**Authors:** Ibrahim A. Akkouh, Silje Skrede, Asbjørn Holmgren, Kari M. Ersland, Lars Hansson, Shahram Bahrami, Ole A. Andreassen, Vidar M. Steen, Srdjan Djurovic, Timothy Hughes

**Affiliations:** 1 NORMENT, Institute of Clinical Medicine, University of Oslo, Oslo, Norway; 2 Department of Medical Genetics, Oslo University Hospital, Oslo, Norway; 3 NORMENT, Department of Clinical Science, University of Bergen, Bergen, Norway; 4 Dr. Einar Martens’ Research Group for Biological Psychiatry, Department of Medical Genetics, Haukeland University Hospital, Bergen, Norway; 5 Division of Mental Health and Addiction, Oslo University Hospital, Oslo, Norway

**Keywords:** Gene expression, Bipolar disorder, RNA sequencing

## Abstract

Lithium has been the first-line treatment for bipolar disorder (BD) for more than six decades. Although the molecular effects of lithium have been studied extensively and gene expression changes are generally believed to be involved, the specific mechanisms of action that mediate mood regulation are still not known. In this study, a multi-step approach was used to explore the transcriptional changes that may underlie lithium’s therapeutic efficacy. First, we identified genes that are associated both with lithium exposure and with BD, and second, we performed differential expression analysis of these genes in brain tissue samples from BD patients (*n* = 42) and healthy controls (*n* = 42). To identify genes that are regulated by lithium exposure, we used high-sensitivity RNA-sequencing of corpus callosum (CC) tissue samples from lithium-treated (*n* = 8) and non-treated (*n* = 9) rats. We found that lithium exposure significantly affected 1108 genes (FDR < 0.05), 702 up-regulated and 406 down-regulated. These genes were mostly enriched for molecular functions related to signal transduction, including well-established lithium-related pathways such as mTOR and Wnt signaling. To identify genes with differential expression in BD, we performed expression quantitative trait loci (eQTL) analysis on BD-associated genetic variants from the most recent genome-wide association study (GWAS) using three different gene expression databases. We found 307 unique eQTL genes regulated by BD-associated variants, of which 12 were also significantly modulated by lithium treatment in rats. Two of these showed differential expression in the CC of BD cases: *RPS23* was significantly down-regulated (*p* = 0.0036, fc = 0.80), while *GRIN2A* showed suggestive evidence of down-regulation in BD (*p* = 0.056, fc = 0.65). Crucially, *GRIN2A* was also significantly up-regulated by lithium in the rat brains (*p* = 2.2e-5, fc = 1.6), which suggests that modulation of *GRIN2A* expression may be a part of the therapeutic effect of the drug. These results indicate that the recent upsurge in research on this central component of the glutamatergic system, as a target of novel therapeutic agents for affective disorders, is warranted and should be intensified.

## Introduction

Lithium has been the treatment of choice for bipolar disorder (BD) for more than six decades [1, 2]. It is effective both as a long-term prophylactic and as an acute treatment for manic and depressive episodes, and approximately one-third of lithium-treated BD patients show excellent response [2, 3]. Although the biological basis of lithium response has been studied extensively, the specific molecular mechanisms mediating its therapeutic effect in BD remain elusive [4]. However, since lithium’s clinical effects typically require several weeks to develop, it is generally assumed that transcriptional changes are involved [5].

The advent of high-throughput gene expression techniques has provided a feasible and bias-free method to screen the whole transcriptome for potential mediators of lithium’s beneficial effects at the molecular level. Since Bosetti et al. published their seminal paper on lithium’s genome-wide transcriptional effects in 2001 [6], numerous studies have examined the gene expression changes produced by lithium, employing different tissues and various model organisms [7]. Despite the low-reproducibility rate exhibited by these transcriptome studies [7], they all point in the same direction: the molecular effects of lithium are highly complex and variegated, involving a multitude of genes and proteins, some with bigger effect sizes than others.

Most of these studies screened the transcriptome using microarray technology, which is known to have several drawbacks, such as a high level of background noise and a narrow dynamic range [8]. RNA-sequencing avoids the technical issues inherent in microarrays because it relies on direct sequencing of transcripts rather than probe hybridization. The superior specificity and broader dynamic range of RNA-sequencing allow for the detection of low-abundance transcripts and more differentially expressed genes with higher fold changes [8]. In addition, RNA-sequencing makes it possible to quantify gene expression at the level of individual transcripts, increasing the resolution and thereby the power to discover distinct isoforms that are differentially regulated. Thus, the full set of lithium’s transcriptional effects can be captured, including subtle gene expression changes.

Given that the clinical effects of lithium are rather specific while its molecular effects are highly heterogenous, it is expected that only a fraction of these molecular effects are responsible for lithium’s mood stabilizing properties [9]. One way to identify the genetic mediators of therapeutic efficacy is to relate the expressional effects of lithium to transcriptional changes of BD-associated genes in human brain samples. The recent publication of the largest BD genome-wide association study (GWAS) [10] provides us with the most extensive list to date of genomic loci that are statistically associated with the disorder. Thus, there is a unique opportunity to further elucidate the intricate relationships between lithium’s molecular and therapeutic effects in BD.

In this study, we used a multi-step approach to search for the transcriptional changes that may underly lithium’s therapeutic efficacy. We first identified genes that are associated both with lithium treatment and with BD, and then we performed differential expression (DE) analysis of these genes in brain tissue samples from BD patients (*n* = 42) and healthy controls (*n* = 42). To identify genes that are regulated by lithium exposure, we used high-sensitivity RNA-sequencing of corpus callosum (CC) tissue samples from lithium-treated (*n* = 8) and non-treated (*n* = 9) rats. To identify genes with DE in BD, we performed expression quantitative trait loci (eQTL) analysis on BD-associated genetic variants from the most recent GWAS using three different gene expression databases. Our main objective was to explore the gene expression changes that potentially mediate the clinical effects of lithium in BD.

## Materials and methods

### Animal handling, lithium treatment, and tissue dissection

All experiments were approved by and carried out in accordance with the guidelines of the Norwegian Committee for Experiments on Animals (Forsøksdyrutvalget, FDU: ID 2015-7661). Female outbred Sprague-Dawley rats (Mollegaard) were kept under standard conditions with an artificial 12:12-hour light/dark cycle (lights on: 08:00) and constant 48% humidity. Animals were housed 5 per cage and allowed access to tap water and free (*ad libitum*) access to standard laboratory chow (Special Diets Services) during the whole experimental period. Care was taken to ensure minimal suffering of the animals at all stages of the experiment. The rats were anesthetized with 2.5% isoflurane gas (Isoba vet; Schering-Plough, Denmark) and Alzet osmotic minipumps (model 2ML4; DURECT Corporation) were implanted according to the manufacturer’s instructions. Rats received either vehicle (*n* = 9) or 2 mmol/kg/day (84.8 mg/kg/day) of lithium chloride (*n* = 8) for 4 days. After deep anesthetization and sacrifice by decapitation, truncal blood was collected in EDTA tubes, and brains were rapidly removed from the skull, briefly washed in ice-cold phosphate buffered saline (PBS), and placed on ice. The whole CC was carefully dissected. Plasma concentrations of lithium were photometrically measured on a Cobas 8000 C702 module (Roche Diagnostics).

### RNA extraction and sequencing

Total RNA was extracted from the rat CC samples with the RNeasy Plus Mini Kit (QIAGEN) according to manufacturer’s protocol. RNA yield was quantified using a NanoDrop 8000 Spectrophotometer (NanoDrop Technologies, Inc.) and RNA integrity was assessed with Bioanalyzer 2100 RNA 6000 Nano Kit (Agilent Technologies, Inc.). Paired-end RNA-seq libraries were prepared with the TruSeq Stranded mRNA kit from Illumina which involves Poly-A purification to capture coding as well as several non-coding RNAs. The prepared samples were sequenced on an Illumina HiSeq 4000 platform (Illumina, Inc.) at an average depth of 50 million fragments per sample using a read length of 150 base pairs and an insert size of 350 base pairs.

### Data processing

Raw sequencing reads were first quality assessed with FastQC (Babraham Institute, Cambridge, UK) and further processed by cutting individual low-quality bases and removing adapter and other Illumina-specific sequences with Trimmomatic V0.32 [11] using default parameters. For the differential gene expression (DGE) analysis, HISAT2 [12] was used to first build a transcriptome index based on ENSEMBL annotations and then to map the trimmed reads to the rat reference transcriptome (Rnor_6.0). To quantify gene expression levels, mapped reads were summarized at the gene level using featureCounts [13] guided by ENSEMBL annotations. For the differential transcript expression (DTE) analysis, RSEM [14] and Salmon [15] were used to estimate transcript expression abundances. The R package *tximport* [16] was used to integrate the transcript-level abundance estimates from both tools into count-based DE analyses engines.

### Estimation of cell type abundances

Cell type abundances were estimated with CIBERSORT v1.06 [17]. On the web interface, 500 permutations were chosen and the quantile normalization option was disabled. The cell types that were considered relevant for CC were astrocytes, endothelial cells, microglia, neurons, and oligodendrocytes. Each of the marker genes was assigned a cell-type specific expression value based on three transcriptome-wide RNA expression murine data sets [18]. Differences in cell type proportions between lithium-treated rats and control rats were assessed with two-sample *t*-tests.

### DE analysis

We applied a pre-filtering step in which genes with less than eight read counts in more than half of the samples were filtered out. The statistical R package *DESeq2* was used for DE analysis [19] using default parameters. *DESeq2* performs an independent filtering step using the means of normalized counts as a filter statistic. A threshold for the filter statistic is found which optimizes the number of adjusted p-values below a user-specified significance level [19]. Using an FDR-adjusted p-value cutoff of 0.05, 14,981 genes were retained in the DGE analysis, and 14854 and 16414 transcripts were retained in the DTE analyses based on transcript quantification with RSEM and Salmon, respectively.

### Pathway enrichment analysis

We performed pathway analysis on the 1051 significant DGE genes with *pathfindR* [20] using a customized pathway reference set for *Rattus norvegicus* based on the Kyoto Encyclopedia of Genes and Genomes (KEGG) database [21]. The R package *clusterProfiler* [22] was used to perform over-representation test on the 57 genes exclusively identified in the DTE analysis and the 112 genes that overlapped with previous transcriptomic reports. The parameters used were: subontology = “BP”, *p*-value cutoff = 0.05, and *q*-value cutoff = 0.05. This over-representation analysis was based on Gene Ontology [23] terms rather than KEGG pathways.

### eQTL analysis of lead SNPs from the PGC bipolar disorder GWAS

eQTL functionality for the 30 BD-associated lead SNPs from the Psychiatric Genomics Consortium’s (PGC) most recent GWAS [10] was examined using publicly available data from the Genotye-Tissue Expression (GTEx) dataset v7 [24], the Brain eQTL Almanac (Braineac) dataset [25], and the CommonMind Consortium (CMC) release 3.0 dataset [26]. Only brain tissues were included in the analyses, and an eQTL association was defined as significant if the FDR value was <0.05. See Supplementary Materials and Methods for a detailed description of the eQTL procedures.

### DGE analysis in human brain samples

Human CC samples from 42 BD patients and 42 healthy controls (Table S16) were obtained through the NIH NeuroBioBank from the Harvard Brain Tissue Resource Center and the University of Pittsburgh Brain Tissue Donation program. The tissues were post-mortem and fully de-identified, and are therefore classified as exempt from Human Subject Research regulations. Total RNA was purified from the samples using TRIzol RNA Isolation reagent according to the manufacturer’s protocol (Thermo Fisher Scientific). cDNA synthesis was performed on 1000 ng of each sample with the High-Capacity cDNA Reverse Transcription Kit (Applied Biosystems, Thermo Fisher Scientific), using 20 μL reaction volume. Gene expression of 12 eQTL genes overlapping with lithium-associated DE genes were investigated using a custom designed TaqMan® Low Density Array with a total of 16 assays (Table S17). Relative expression levels were calculated using the ∆∆Ct method [27], normalizing each gene of interest against the mean cycle threshold (Ct) of four endogenous control genes (Table S17). For each of the 12 lithium-associated genes, a DE analysis between BD and CTRL subjects was performed using a simple logistic regression model in R adjusting for sex and age differences. *p*-values were not corrected for multiple testing.

## Results

### Pre-analysis assessments

**Fig. 1:**
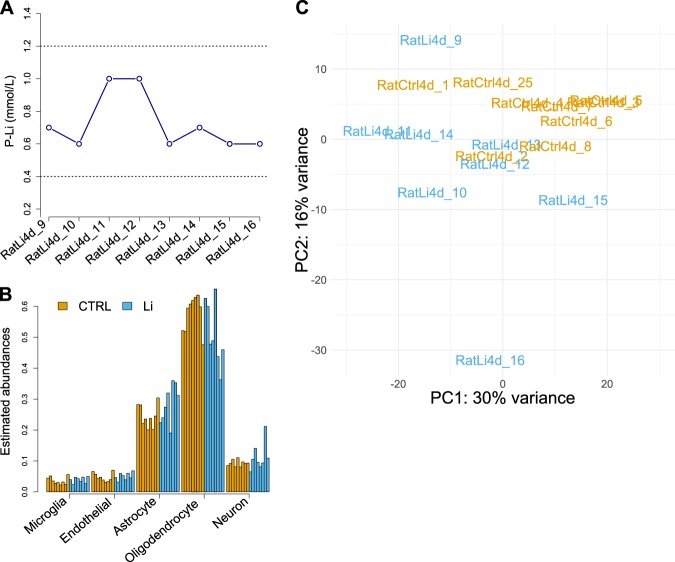
Initial assessments of lithium treatment and sequencing results. **a** All lithium-treated rats had plasma concentrations of lithium chloride between 0.6–1.0 mmol/L, which is well within therapeutically relevant concentrations in humans as indicated by the two dotted lines. **b** Bar graphs showing the estimated cell type abundances (in percentage) for five relevant cell types as determined by cell type deconvolution analysis. Each bar represents a single rat sample. **c** PCA plot showing good separation on condition over the first two principal components, explaining 46% of the variation in total. The plot also revealed an outlier sample, which was excluded from the analysis. The PCA analysis was based on expression data from all the ~15000 genes that survived the pre-filtering steps

To ensure a controlled administration of lithium, subcutaneous minipumps were used to continuously administer lithium chloride into 8 female rats at a total dose of 2 mmol/kg/day. After 4 days of treatment, serum lithium levels in all treated rats were between 0.6 and 1.0 mmol/L (mean = 0.725, sd = 0.175) (Fig. 1a). These levels are close to the optimal lithium concentration in humans, and well within therapeutically relevant levels for the treatment of BD [28, 29].

Since the CC primarily consists of myelinated axons, we expected that the majority of mRNAs would come from oligodendrocytes. Using the computational cell type deconvolution tool CIBERSORT [17], we estimated the abundances of five relevant cell types. We found that the two most predominant cell types were oligodendrocytes and astrocytes, with estimated proportions of ~55% and ~25%, respectively (Fig. 1b). Differences in cell type proportions are a major source of variation in gene expression studies, potentially accounting for up to 13% of the observed expression discrepancy between conditions [30]. Using two-sample *t*-tests to examine the cell type proportions in treated and untreated rats, we found no significant differences in any of the assessed cell types (Fig. 1b). Coupled with the fact that all rats were consistently kept under equal experimental settings, this finding suggests that most of the observed gene expression variation can be attributed to lithium’s molecular effects rather than other confounding factors.

Principal component analysis (PCA) of the full set of expressed genes revealed a clear but incomplete clustering by condition along the two first principal components, explaining 46% of the total variance (Fig. 1c). The PCA analysis also revealed that one of the treated rats, RatLi4d_16, had a distinct expression profile separating it from the rest of the samples (Fig. 1c). This sample was thus considered an outlier and excluded before conducting the DE analyses.

### DGE analysis

**Fig. 2:**
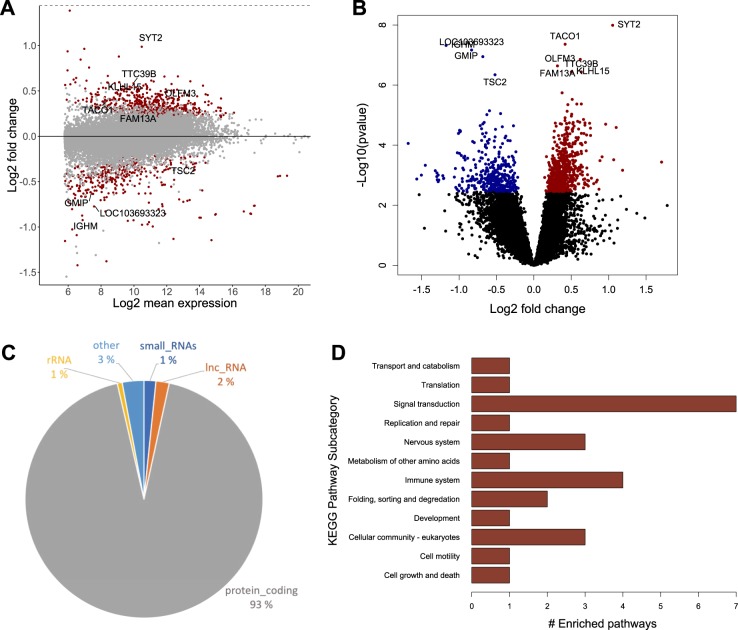
Differential gene expression (DGE) analysis of CC samples from lithium-treated and untreated rats. **a** MA-plot showing the relationship between mean expression values and fold changes for all analyzed genes. Each dot represents a gene. Significantly associated genes (FDR < 0.05) are colored in red, and the 10 most significant genes (lowest FDR value) are labeled. As the plot shows, the effect size variance is dependent on the mean expression value. Genes with lower average expression tend to have bigger fold changes between conditions, indicating the increased uncertainty in effect size estimates of low-abundance genes and the need for pre-filtering. **b** Volcano plot showing the fold change and *p*-value for each gene. Genes with significant up-regulation (FDR < 0.05) in lithium-treated rats are colored in red, and genes with significant down-regulation are colored in blue. The 10 genes with the lowest *p*-values are labeled. **c** Biotypes of lithium-associated DGE genes. The majority of genes were protein coding. Small RNAs include miRNAs and snoRNAs. “Other” include pseudogenes and processed pseudogenes. lncRNA: Long non-coding RNA. **d** Grouping of significantly enriched DGE pathways according to the KEGG subcategory arrangement. Most of the enriched pathways were involved in signal transduction processes (*n* = 7) and immune system functions (*n* = 4). KEGG: The Kyoto Encyclopedia of Genes and Genomes

To identify genes with expression levels significantly altered by exposure to lithium, we first performed a genome-wide differential expression analysis on the gene-level (DGE) by counting aligned RNA-sequencing reads that overlap with gene regions. Quality control and alignment metrics of the sequencing reads can be found in the supplementary materials (Figs. S1–S4). The DGE analysis resulted in 1051 significant differentially expressed genes, 669 up-regulated and 382 down-regulated (Fig. 2a, b, Table S1–S2). The majority of identified genes were protein coding (93%), but there were also a number of non-coding RNA genes (Fig. 2c and Fig. S5). Effect sizes (fold changes) ranged from 0.31 to 3.27, with the most down-regulated protein coding gene being *ATP14* (fc = 0.40, *p* = 7.07e-4) and the most up-regulated gene being *GPR101* (fc = 3.27, *p* = 3.66e-4).

### Assessing the role of CC and oligodendrocytes

Since the CC in general and oligodendrocyte dysfunction in particular have been implicated in the pathophysiology of BD [31–33], a partial aim of the current study was to investigate whether the molecular effects of lithium are mediated by mechanisms underlying myelination and other oligodendrocyte-specific functions. We examined whether our list of 1051 DGE genes overlapped with curated sets of myelin-related [34] and oligodendrocyte-specific genes [35], and found that lithium significantly modulated 7 and 6 genes, respectively (Table S4). One of these genes, *MBP* (Myelin basic protein) is especially relevant as the encoded product is the second most abundant myelin-related protein constituting around 30% of total myelin protein [32]. However, none of the gene sets were significantly over-represented in our samples (Fisher’s exact test: p(myelin) = 0.84, *p*(oligo) = 0.48).

### Pathway analysis of DGE genes

A common way to reduce complexity of analysis in high-throughput experiments is to perform pathway analysis, which aims to increase explanatory power by grouping long lists of genes into smaller sets of genes or proteins that function in the same cellular pathways. By conducting pathway analysis on our list of 1051 DGE genes, we identified 26 pathways that were significantly enriched by lithium exposure in the rat CC (Fig. S6, Table S5–S6). Utilizing the subcategory arrangement of the Kyoto Encyclopedia of Genes and Genomes (KEGG) database to group the enriched pathways into broader cellular processes, we found that the primary effects of lithium were related to signal transduction and immunological functions, but there were also clear effects on nervous system and cellular community processes (Fig. 2d). Moreover, most of the significantly enriched pathways were activated (up-regulated) by lithium (Fig. S7).

### DTE analysis

**Fig. 3:**
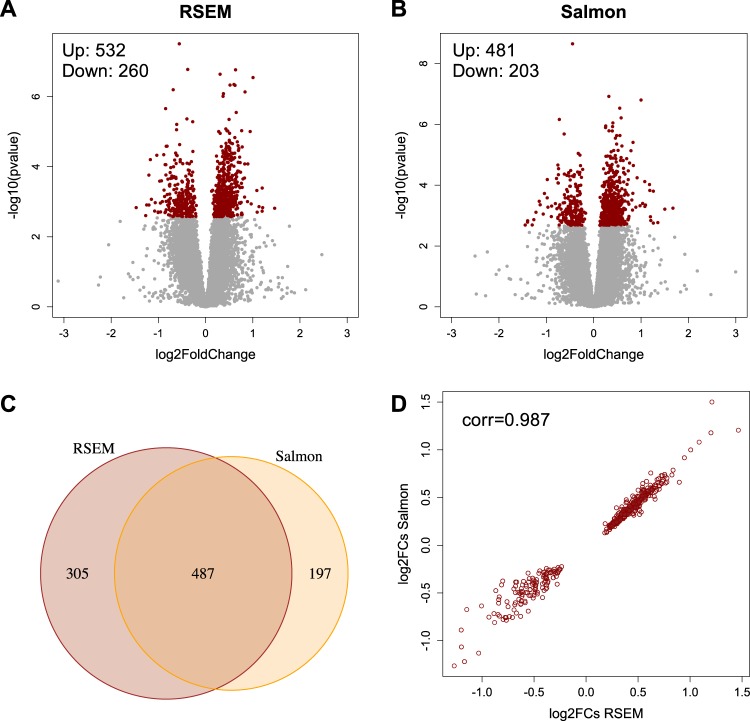
Differential transcript expression (DTE) analysis of CC samples from lithium-treated and untreated rats. The volcano plots depict the fold changes and *p*-values for all analyzed transcripts based on expression quantification with **a** RSEM and **b** Salmon. Significant differentially expressed transcripts (FDR < 0.05) are colored in red. **c** Venn diagram of significant DE transcripts as determined with RSEM and Salmon quantification, respectively. The intersection represents the number of transcripts (*n* = 487) that were identified as DE with both tools. **d** The 487 DE transcripts commonly identified with RSEM and Salmon quantification had highly correlated fold changes (*r* = 0.987) in terms of both the magnitude and direction of effect. RSEM: RNA-seq by expectation maximization

Although gene-level DE analysis can be more robust than its transcript-level counterpart, it can also mask transcript-level dynamics [36]. Moreover, transcript-level analysis provides higher resolution than gene count-based approaches [16]. For these reasons, we performed a DTE analysis to discover additional lithium-associated genes that were not identified in the DGE analysis. DTE analysis based on expression levels estimated with RSEM and Salmon resulted in 792 and 684 differentially expressed genes, respectively (Fig. 3a, b, Tables S7–S8). We found a high degree of overlap (487 DTE genes) between the two tools (Fig. 3c, Table S9), and the overlapping genes were in almost complete concordance with respect to both the magnitude and direction of the effect sizes (Fig. 3d). Of these 487 significant DTE genes, 430 (88.3%) were also identified in the DGE analysis with similar *p*-values and fold changes (Fig. S8). This similarity is most probably due to the fact that the majority of the 430 shared genes have only one transcript, yielding highly similar results with both gene-level and transcript-level methods. The DTE analysis also identified 57 DTE genes, 33 up-regulated and 24 down-regulated, that were not detected in the DGE analysis (Fig. S8A, Table S10). These DTE-only genes were enriched for GO terms related to neuronal processes (Fig. S9, Table S11).

### Comparison between identified genes and previous findings

To relate our results to previous lithium findings, we compared the full set of DGE and DTE-only genes (*n* = 1108) with a set of genes associated with lithium in 18 previous transcriptomic reports [37–54] identified through a systematic literature search (Supplementary Materials and Methods). We found overlap with 112 genes (10.1%), many of them having similar direction and magnitude of effect (Table S3). These genes were enriched for biological processes related to cytoplasmic protein translation (*p* = 1.29e-5, fc = 9.00).

### DE analysis of BD-associated lithium genes in human brain samples

**Fig. 4:**
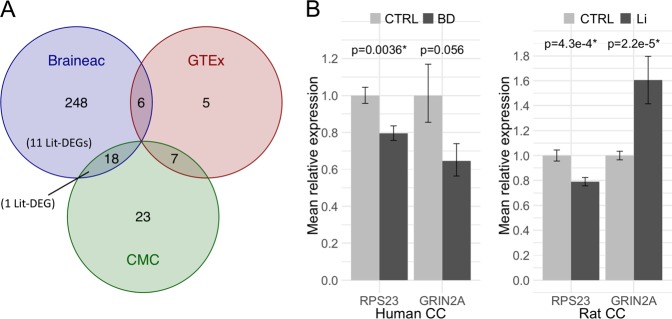
Expression of lithium-associated eQTL genes in human brain samples. **a** Venn diagram showing the number of BD-associated eQTL genes identified using each of the three gene expression databases Braineac, GTEx, and CMC. Intersections show the number of eQTL genes shared between databases. No single eQTL gene was identified by all three databases. 11 of the 12 genes that were both lithium-associated DE genes (Lit-DEG) and BD-associated eQTL genes were identified only by Braineac, while one gene was identified by both Braineac and CMC. **b** Bar plots depicting the expression levels of *RPS23* and *GRIN2A* in human CC samples (left) and rat CC samples (right). The error bars show the standard errors for the mean relative expressions. eQTL: expression quantitative trait locus, Braineac: the brain eQTL Almanac, GTEx: genotype-tissue expression, CMC: The CommonMind Consortium, Lit: lithium, DEG: differentially expressed gene, CTRL: healthy human control subjects, BD: bipolar disorder, CC: Corpus callosum

To elucidate the relationship between lithium’s molecular and therapeutic effects in BD, the disease-associated lead SNPs from the largest and most recent BD GWAS [10] were screened for expression quantitative trait (eQTL) effects in brain tissue gene expression data from three publicly available gene expression databases: Braineac, gTEX, and CMC (Supplementary Materials and Methods). Separate eQTL analyses resulted in the identification of 272, 18, and 48 eQTL genes in the three databases, respectively (Tables S12–S14). No eQTL gene was identified by all three databases, but 18 genes were shared between Braineac and CMC, six genes were shared between Braineac and gTEX, and seven genes were shared between gTEX and CMC (Fig. 4a). Of the 307 unique eQTL genes identified in total, 12 overlapped with the lithium-associated genes (Fig. 4a, Table S16). Expression levels of these 12 genes, which were associated with both lithium exposure in the rat CC and with BD, were assessed in human post-mortem CC samples from 42 BD patients and 42 healthy controls. The *RPS23* gene was significantly downr-egulated in BD cases (*p* = 0.0036), with 20% reduced expression relative to controls (Fig. 4b, Table S16). The direction of gene expression regulation in BD was the same as in lithium exposure. In addition, a suggestive association with BD was found for *GRIN2A* (*p* = 0.056), which had a 35% reduction in expression levels compared to healthy controls (Fig. 4b, Table S16). Importantly, this effect was in the opposite direction of what we found in the rat CC, where expression of *GRIN2A* was significantly up-regulated by lithium exposure (*p* = 2.2e-5, fc = 1.6; Fig. 4b).

## Discussion

In the present study, we employed deep RNA-sequencing at both gene and transcript levels to perform a high-sensitivity characterization of the gene expression changes induced by lithium exposure in the rat CC, which is a brain structure consisting almost exclusively of myelinated axons and is responsible for communication between the two hemispheres. The CC in general and myelinating oligodendrocytes in particular have been implicated in the etiology of BD and in lithium’s mechanisms of action [31–33, 55–59]. The main objective of the study was to explore the transcriptional effects that may mediate lithium’s therapeutic efficacy by relating lithium findings to BD-associated genes and measuring their expression levels in post-mortem CC samples from BD patients and healthy controls. A partial aim was to investigate whether the CC and oligodendrocytes play a central role in the drug’s mechanisms of action. The DGE analysis resulted in 1051 significant DE genes, 669 up-regulated and 382 down-regulated. Most of these genes were related to signal transduction and immunological functions. In addition, 57 genes were identified exclusively in the DTE analysis. These genes were enriched for neuronal processes. Of the total set of 1108 lithium-associated genes, 12 were also associated with BD based on eQTL analyses of BD-associated lead SNPs from the most recent GWAS. Of these 12 BD and lithium-related genes, *RPS23* was significantly down-regulated in brain samples from BD patients, while *GRIN2A* had a near to significant down-regulation in BD.

The full set of lithium-associated genes identified in the present study is relatively large compared to other transcriptional studies, most of which measured gene expression using microarray technology. The extensive number of hits can to a large extent be attributed to the fact that expression levels were assessed with high-coverage RNA-sequencing which widens the dynamic range and thereby increases both sensitivity and specificity. The sensitivity was further improved by performing a transcript-level analysis which allowed us to identify 57 additional DE genes, demonstrating one of the benefits of analyzing individual transcripts as a complementary strategy to the gene-level approach. Approximately 10% of the genes identified here have been linked to lithium exposure in previous studies (Table S3). Although this is a relatively poor replication rate, it is not unexpected given the general lack of replicated genetic findings in lithium studies [7, 60]. One reason for the observed discrepancy between studies may be that different tissues and organisms are deployed, and that different methodological and statistical approaches are followed. Nevertheless, it is interesting to note that one of the genes, *RRAGC*, was implicated in 3 other studies, two of which analyzed human lymphoblastoid cell lines and one that analyzed mouse brain. Moreover, the effect size of *RRAGC* was comparable in all studies, including our own (Table S3). The protein encoded by *RRAGC* (Ras-related GTP binding C) promotes intracellular localization of the mammalian target of rapamycin (mTOR) complex, which plays a central role in cellular growth and metabolism [61].

The picture emerging from pharmacological and genetic studies is that signal transduction pathways play a central role in the mechanisms of action of lithium [9]. In addition to the long-standing and widely discussed inositol depletion hypothesis [62], lithium has been shown to modulate glycogen synthase kinase 3 (GSK-3), protein kinase C (PKC), mTOR, and Wnt signaling among others [4]. Our finding that signal transduction pathways were the most affected by lithium exposure is in line with the convergent evidence. These findings included already established associations like mTOR and Wnt signaling, but also interesting candidates such as the ErbB, MAPK, and VEGF pathways [63]. The potential immunoregulatory effects of lithium were proposed two decades ago [64], and recent evidence has shed new light on this relationship [37]. This aspect of lithium pharmacogenetics seems especially relevant given the strong implication of immune system dysfunction in BD [65, 66]. We found that lithium regulated several immune-related pathways, including B-cell receptor, T-cell receptor, and chemokine signaling pathways, providing further support for lithium’s effect on components of the immune system.

A partial aim of the current study was to investigate whether lithium specifically targets the CC and oligodendrocytes, as these have been implicated in both BD and lithium action. Comparing our set of lithium-associated genes with curated lists of myelin-related and oligodendrocyte-specific genes, we found no significant enrichment. The way we interpret these results is (a) that myelination is probably not a central target of lithium’s mechanisms of action in the brain, and (b) that the molecular effects of lithium in the CC and oligodendrocytes are not different from its effects in other brain regions and cell types. Indeed, as most molecular studies have found that lithium has widespread effects on gene expression regardless of the tissue or cell type investigated, it seems likely that the molecular effects of lithium are systemic rather than targeted to specific cell types or brain areas.

Although the molecular effects of lithium appear to be highly variegated and complex, it is expected that only a subset of these are directly involved in the drug’s therapeutic properties. Linking lithium-affected genes in the rat brain to genetic variants associated with BD may help to elucidate the intricate relationship between BD and lithium’s therapeutic efficacy. We found that the expression levels of 12 genes were modulated by both lithium exposure and functional variants associated with BD (Fig. 4a, Table S15). Only *RPS23* was significantly differentially expressed in brain samples from BD subjects, but the effect was in the same direction as the effect of lithium, which is inconsistent with what one would expect if this gene was a mediating factor in lithium’s therapeutic effects. *RPS23*, ribosomal protein S23, encodes a protein component of the small subunit of the ribosomal complex, and has been implicated in several developmental disorders [67]. DE analysis in human brain samples also revealed a suggestive finding in the *GRIN2A* gene, which had reduced expression in BD patients (Fig. 4b). *GRIN2A* was strongly up-regulated by lithium exposure in rats and may thus provide an interesting link between the molecular and therapeutic effects of lithium. The protein encoded by *GRIN2A*, GluN2A, forms a modulatory subunit of a subset of *N*-Methyl-D-aspartate (NMDA) receptors, which are calcium channels gated by the major excitatory neurotransmitter glutamate.

The present study has several strengths, such as the implementation of a robust methodological and analytical approach that combines animal experiments, RNA-sequencing, eQTL analysis, and gene expression assessments in human post-mortem brain samples. On the other hand, it also has some important limitations. First, rats were exposed to lithium for four days, which is sufficient to reach equivalent concentrations in blood and brain [68], but the results cannot be extrapolated to a long-term clinical context where consequences of primary molecular effects cannot be distinguished from subsequent feedback responses. Our aim was to explore the immediate transcriptional effects of lithium, but since these initial changes may eventually lead to secondary homeostatic adjustments if given longer time, future experiments should include rats exposed to long-term treatment with lithium. Second, information on pharmacological treatment and clinical response for the brain donors was not available, and these factors could therefore not be taken into account in the DE analysis. Furthermore, an underlying assumption of the study design is that the susceptibility genes for BD are also those that are most likely involved in lithium’s mood stabilizing properties. Although the validity of this assumption remains unproven, we believe it is a plausible and justified assumption, as it seems unlikely that none of the molecular effects of lithium are related to the variants associated with BD. Further, the increasing sample sizes of BD GWASs, culminating in the most recent study by the PGC [10], mean that they are sufficiently powered to robustly identify variants that are involved in the etiology of the disease. It should be noted, however, that even if BD-associated genes were shown to be involved, the implemented approach still lacks the ability to separate lithium’s molecular effects from its therapeutic effects. This can only be achieved by comparing well-characterized lithium responders and non-responders. Thus, although the present study identifies two genes with potential therapeutic relevance, these findings should be corroborated by findings from well-powered lithium response GWASs.

With these considerations in mind, the suggestive association with *GRIN2A* is particularly interesting. Signaling through NMDA receptors is critical for normal development, learning, memory, and other cognitive functions [69]. More than 60 different mutations of *GRIN2A* have been found in patients presenting with epilepsy-aphasia spectrum disorders, intellectual disability, and Parkinson’s disease [69]. Disturbance of the glutamatergic system has also been consistently implicated in affective disorders like major depression and BD, and has recently received increasing attention as a potential target of new therapeutic agents for mood disorders [70, 71]. Given the documented effects of lithium on intracellular calcium modulation in general, the association with *GRIN2A* may point to a potential role for this gene in lithium’s positive effects in BD. Our results indicate that the recent upsurge in research on this central component of the glutamatergic system as a potential therapeutic target is warranted and should be intensified.

## Funding and disclosure

The study was supported by funding from the South-East Norway Regional Health Authority (2018013 and 2018094) and the Research Council of Norway (223273). OAA has received a speaker’s honorarium from H. Lundbeck AS. All other authors declare no conflict of interest.

## Supplementary information

Supplementary Methods

Supplementary Figures S1 and S2

Supplementary Figures S3 and S4

Supplementary Figures S5 and S6

Supplementary Figures S7 and S8

Supplementary Figure S9

Supplementary Tables
