# Supplementary material for: Exploring lithium’s transcriptional mechanisms of action in bipolar disorder: a multi-step study
Source: Neuropsychopharmacology. 2019 Oct 25;45(6):947–55. doi: 10.1038/s41386-019-0556-8 (PMC7162887; doi:10.1038/s41386-019-0556-8)
Supplement: Supplementary file 1 — Supplementary Methods [file 41386_2019_556_MOESM1_ESM.pdf]

**SUPPLEMENTARY INFORMATION**

# Exploring Lithium's Transcriptional Mechanisms of Action in Bipolar Disorder: A Multi-Step Study

Ibrahim A. Akkouh, Silje Skrede, Asbjørn Holmgren, Kari M. Ersland, Lars Hansson, Shahram Bahrami, Ole A. Andreassen, Vidar M. Steen, Srdjan Djurovic, Timothy Hughes

## SUPPLEMENTARY MATERIALS AND METHODS

### Animal handling and lithium treatment

All experiments were approved by and carried out in accordance with the guidelines of the Norwegian Committee for Experiments on Animals (Forsøksdyrutvalget, FDU: ID 2015-7661). Female outbred Sprague-Dawley rats (Mollegaard, Denmark) were kept under standard conditions with an artificial 12:12-hour light/dark cycle (lights on: 08:00) and constant 48% humidity. Animals were housed 5 per cage and allowed access to tap water and free (*ad libitum*) access to standard laboratory chow (Special Diets Services, Witham, UK) during the whole experimental period. Care was taken to ensure minimal suffering of the animals at all stages of the experiment. Lithium chloride (Merck, Darmstadt, Germany) was dissolved in sterile water (Lonza, Verviers, Belgium). Rats were anesthetized with 2.5% isoflurane gas (Isoba vet; Schering-Plough, Denmark) and Alzet osmotic minipumps (model 2ML4; DURECT Corporation, Cupertino, CA, USA) were implanted according to the manufacturer's instructions. Rats received either vehicle (n=9) or 2 mmol/kg/day (84.8 mg/kg/day) of lithium chloride (n=8) for 4 days.

### Tissue dissection and measurement of lithium concentrations

Rats were deeply anesthetized by isoflurane and subsequently sacrificed by decapitation. Truncal blood was collected in EDTA tubes and centrifuged at 3000 g for 10 minutes (4° C) in order to extract plasma, which was stored at -80°C. Brains were rapidly removed from the skull, briefly washed in ice-cold phosphate buffered saline (PBS), and placed on ice. The whole corpus callosum (CC) was carefully dissected. In addition, tissue samples from the following regions of the right hemisphere were harvested: fronto-medial cortex (FMCx), temporal cortex, occipital cortex, striatum, hippocampus and cerebellum. All tissue samples were immediately flash frozen on dry ice and subsequently stored at -80°C. Plasma concentrations of lithium were photometrically measured on a Cobas 8000 C702 module (Roche Diagnostics, Indianapolis, USA).

### RNA extraction and sequencing

Frozen CC samples were submerged in pre-chilled Ambion RNAlater-ICE Frozen Tissue Transition Solution (Thermo Fisher Scientific) and allowed to thaw overnight at -20°C. Total

RNA was extracted with the RNeasy Plus Mini Kit (QIAGEN) according to manufacturer's protocol. RNA yield was quantified using a NanoDrop 8000 Spectrophotometer (NanoDrop Technologies, Inc.) and RNA integrity was assessed with Bioanalyzer 2100 RNA 6000 Nano Kit (Agilent Technologies, Inc.) in accordance with manufacturer's instructions. All samples had a RIN value above 9.5. Prior to RNA-sequencing, 2 µL of a 1:100 dilution of ERCC RNA Spike-In Mix (Thermo Fisher Scientific) were added to each purified total RNA sample. Library preparation and paired-end RNA-sequencing were carried out at the Norwegian High-Throughput Sequencing Centre (www.sequencing.uio.no). Briefly, libraries were prepared with the TruSeq Stranded mRNA kit from Illumina which involves Poly-A purification to capture coding as well as several non-coding RNAs. The prepared samples were then sequenced on an Illumina HiSeq 4000 platform (Illumina, Inc.) at an average depth of 50 million reads per sample using a read length of 150 base pairs and an insert size of 350 base pairs.

**Data processing**

Raw sequencing reads were quality assessed with FastQC (Babraham Institute, Cambridge, UK). To pass the initial QC check, the average Phred score of each base position across all reads had to be at least 30. Reads were further processed by cutting individual low-quality bases and removing adapter and other Illumina-specific sequences with Trimmomatic V0.32 [1] using default parameters. Since the trimming process may result in some reads being discarded and their mates thereby unpaired, only reads that remained paired after trimming were used for downstream analyses. For the differential gene expression (DGE) analysis, HISAT2 [2] was used to first build a transcriptome index based on ENSEMBL annotations and then to map the trimmed reads to the rat reference transcriptome (Rnor_6.0). To quantify gene expression levels, mapped reads were summarized at the gene level using featureCounts [3] guided by ENSEMBL annotations.

For the differential transcript expression (DTE) analysis, two tools, RSEM [4] and Salmon [5], were used to estimate transcript expression abundances. Both tools were shown to perform better than other packages in a recent comprehensive evaluation of computational tools for RNA-seq isoform quantification [6]. In the present study, RSEM was run in the default mode in which raw sequencing reads are provided as input and RSEM itself aligns the reads to the transcriptome reference before transcript expression levels are estimated. STAR was specified as the alignment tool. Salmon can be operated in either a quasi-mapping based mode, in which

raw reads are processed directly, or in an alignment-based mode which takes aligned reads as input [5]. Since the latter mode was shown to perform better than the former [6], the reads already aligned with HISAT2 were provided as input for the alignment-based approach. Both RSEM and Salmon quantification methods were based on ENSEMBL transcript annotations. Finally, the R package *tximport* [7] was used to integrate the transcript-level abundance estimates from both tools into count-based differential expression (DE) analyses engines.

**Estimation of cell type abundances**

Cell type abundances were estimated with CIBERSORT v1.06 [8]. On the web interface, 500 permutations were chosen and the quantile normalization option was disabled. CIBERSORT uses a computational approach to characterize the cell type composition of each sample based on the gene expression profiles of a group of reference cell types. The reference file was constructed by selecting a set of cell-type specific marker genes as defined by Mancarci et al. [9]. The cell types that were considered relevant for the CC region were astrocytes, endothelial cells, microglia, neurons, and oligodendrocytes. Each of the marker genes was assigned a cell-type specific expression value based on three transcriptome-wide RNA expression murine data sets generated within the past several years [10]. Since it has been demonstrated that neuronal axons contain hundreds of mRNA transcripts [11], axonal marker genes were excluded from the reference file in order to avoid an inflated abundance estimate of neurons. To ensure that the estimated cell type abundances were reliable, CIBERSORT was also run on seven pure astrocyte samples differentiated from human induced pluripotent stem cells. The same parameters and reference were used in the validation run. Differences in cell type proportions between lithium treated rats and control rats were assessed with two-sample t-tests.

**Differential expression (DE) analysis**

One of the lithium-treated rats, RatLi4d_16, was identified as an outlier based on visual inspection of a principal component analysis (PCA) plot and excluded prior to the DE analyses. We applied a pre-filtering step in which genes with less than 8 read counts in more than half of the samples were filtered out. The threshold of 8 read counts was determined as the lower limit of detection by comparing the read counts generated from the ERCC RNA spike-ins with their known concentrations (Supplementary Figure S2A). The statistical R package DESeq2 was used for DE analyses [12]. This tool provides methods to test for differentially expressed genes

or transcripts by use of negative binomial generalized models. The DESeq2 workflow begins by taking raw read count data as input and applies an internal normalization method that corrects for sequencing depth and RNA composition. After the default DE analysis, DESeq2 performs an independent filtering step using the means of normalized counts as a filter statistic. A threshold for the filter statistic is found which optimizes the number of adjusted p-values below a user-specified significance level [12]. Using an FDR-adjusted p-value cutoff of 0.05, 14981 genes were retained in the DGE analysis, and 14854 and 16414 transcripts were retained in the DTE analyses based on transcript quantification with RSEM and Salmon, respectively. The final output of the DESeq2 pipeline is a table giving the base mean of normalized counts across samples, log2 fold changes, standard errors, test statistics, raw p-values, and adjusted p-values.

**Identification of replicated findings**

To identify which of the total of 1108 significant DE genes (based on both DGE and DTE analyses) had been implicated in lithium action in previous reports, a systematic literature search in PubMed was conducted. The following combination of search terms and Boolean operators was used: (lithium[Title]) AND (*express*[Title] OR *regulat*[Title]) AND (bipolar[Title/Abstract]) NOT (review[Filter]). This initial search resulted in 89 studies. To further filter the search, only studies that examined the genome-wide effects of lithium on gene expression in mammals were considered relevant, which resulted in 13 studies meeting these criteria. In addition, five more studies that were not identified by the search but that were known by the authors were also considered eligible. In total, 18 reports were screened for replicated findings (references 32-49 in the main article). We did not restrict the search to a specific organism or a specific tissue or cell type as we wanted the assessment to be as comprehensive as possible.

**Pathway enrichment analysis**

Genome-wide DE analyses typically result in a long list of genes that are differentially expressed between conditions. Pathway enrichment analysis is a common way to reduce complexity by grouping the genes into biological themes that facilitate interpretation. We performed pathway analysis on the 1051 DE genes identified in the DGE analysis using pathfindR [13]. Rather than treating all genes equally as many pathway analysis tools do,

pathfindR takes into account both the p-values and effect sizes of individual genes. It also incorporates information on protein-protein interactions between genes and thereby avoids the assumption that each gene is independent from the other genes. Since pathfindR does not yet provide inbuilt functionalities for analyzing non-human gene sets directly, we constructed a customized pathway reference set for *Rattus norvegicus* based on the Kyoto Encyclopedia of Genes and Genomes (KEGG) database [14]. In this custom pathway set, we excluded pathway terms related to human diseases and drug development as we were primarily interested in the molecular mechanisms and biological processes that the identified DE genes are involved in. The 57 genes exclusively identified in the DTE analysis did not result in any significantly enriched pathways when analyzed with pathfindR. Therefore, we used a simpler over-representation analysis tool, clusterProfiler [15], to analyze these genes. The parameters used were: subontology = "BP", p-value cutoff = 0.05, and q-value cutoff = 0.05. This over-representation analysis was based on Gene Ontology (GO) terms [16] rather than KEGG pathways. The same analysis was applied to the 112 lithium-related genes that had been implicated in one or more previous transcriptomic reports. Fold change of the enriched GO term was calculated by the observed gene ratio for the GO term by the background ratio.

**eQTL analysis of lead SNPs from the PGC bipolar disorder GWAS**

Expression quantitative trait loci (eQTL) functionality for the 30 BD-associated lead SNPs with genome-wide significance identified in the Psychiatric Genomics Consortium's (PGC) most recent GWAS [17] was examined using publicly available data from the Genotye-Tissue Expression (GTEx) dataset v7 [18], the Brain eQTL Almanac (Braineac) dataset [19], and the CommonMind Consortium (CMC) release 3.0 dataset [20]. Although all 30 lead SNPs were used as input for the eQTL analyses, only the lead SNPs for which data were available in each database were considered. Thus, 24, 20, and 15 of the lead SNPs were analyzed in GTEx, Braineac, and CMC, respectively,

The GTEx v7 dataset comprises 714 donors, of which 620 had eQTL data available. Participants were genotyped using whole-genome sequencing. Pre-calculated eQTLs were generated by FastQTL [21] for tissues having more than 70 samples, using a +/- 1 Mb *cis* window around the transcript start site. Only brain tissues (n=13) were considered in the present study. Beta distribution-adjusted empirical p-values from FastQTL were used to calculate q-values, and an FDR threshold of ≤0.05 was applied to identify genes with a significant eQTL

association. Nominal p-values were generated for each variant-gene pair by testing the alternative hypothesis that the slope of a linear regression model between genotype and expression deviates from 0. The effect size of the eQTLs was defined as the slope of the linear regression, and was computed as the effect of the alternative allele relative to the reference allele in the human genome reference GRCh37/hg19 (i.e., the eQTL effect allele is the alternative allele). See the original GTEx publication for full details [18].

The Braineac dataset contains genotype and expression data for 10 different brain regions (plus average-all) from brain samples obtained from 134 neurologically normal individuals of European descent. All samples were genotyped on the Illumina Infinium Omni1-Quad BeadChip and on the Immunochip. RNA expression levels were quantified with Affymetrix microarrays. The eQTL analysis was run for each expression profile against every genetic marker in every tissue (plus average-all) using the R package Matrix eQTL [22], which tests the linear model of genotype against normalized expression values using standard asymptotic methods that are equivalent to the usual likelihood ratio test for linear models. An eQTL signal was considered to be *cis*-acting if the marker was located within 1Mb of the transcription start site of the associated transcript. The number of tests conducted for each type of analysis (*cis* or *trans*, SNP or indel etc.) in each tissue (plus average-all) were calculated and used as the basis for converting the nominal p-values into FDRs using the Benjamini-Hochberg procedure [23]. In the present study, an eQTL association was defined as significant if the FDR was <0.05 for the average-all tissue type. More details can be found in the original publication [19].

The CMC 3.0 collection consists of RNA sequencing data and SNP genotypes of the dorsolateral prefrontal cortex (DLPFC), anterior cingulate cortex (ACC), and superior temporal gyrus (STG) from 986 donors comprising the diagnostic categories schizophrenia (n=353), bipolar/affective disorder (n=57), other (n=75), and healthy controls (n=501). Postmortem tissue from schizophrenia and bipolar disorder cases were included if they met the diagnostic criteria in DSM-IV for schizophrenia or schizoaffective disorder, or for bipolar disorder. Eight samples were classified as affective disorder other than bipolar post sample collection. Paired-end RNA-sequencing was performed on a HiSeq 2500, and genotyping was conducted on the Illumina Infinium HumanOmniExpressExome 8 v1.1b chip using the manufacturer's protocol. Gene-level eQTL were computed using a linear model on the imputed genotype dosages using Matrix eQTL [22]. FDR was estimated separately for *cis*-eQTL (defined as <1Mb between SNP marker and gene position) and *trans*-eQTL (>1 Mb between marker and gene position,

controlling for FDR one chromosome at a time. A detailed description of the full pipeline from sample preparation to statistical analysis can be found in Fromer et al. [20].

## Differential gene expression analysis in human brain samples

Post-mortem CC samples from 42 BD patients and 42 healthy controls were obtained through the NIH NeuroBioBank from the Harvard Brain Tissue Resource Center and the University of Pittsburgh Brain Tissue Donation program (see Supplementary Table S16 for sample details). RNA was purified with TRIzol RNA Isolation reagent according to the manufacturer's protocol (Thermo Fisher Scientific). Briefly, 30-100 mg frozen tissue was added to 1 mL ice-cold TRIzol reagent and homogenized with a TissueRuptor II (Qiagen). The lysates were centrifuged for 5 minutes at 12,000 × g at 4°C, and the supernatants transferred to new tubes. After incubation for 5 minutes, 0.2 mL chloroform (Sigma-Aldrich) was added, and the samples were incubated at room temperature for 2-3 minutes and centrifuged for 15 minutes at 12,000 × g at 4°C. The aqueous phase was transferred to a new tube, and 0.5 mL isopropanol (Sigma-Aldrich) was added. The samples were incubated at room temperature for 10 min, followed by centrifugation at 12,000 x g for 10 min at 4 °C. The pellet was washed with 75 % Ethanol (Sigma-Aldrich), air-dried and resuspended in 20-50 µL nuclease-free water (Qiagen). The RNA was further purified with RNeasy MinElute Cleanup Kit (Qiagen), and eluted in 20 µL nuclease-free water. RNA concentration of each sample was measured on a NanoDrop 1000 spectrophotometer (Thermo Fisher Scientific), and integrity of the RNA was assessed on an Agilent 2100 Bioanalyzer using RNA 6000 Nano chips (Agilent Technologies). cDNA synthesis was performed on 1000 ng of each sample with the High-Capacity cDNA Reverse Transcription Kit (Applied Biosystems, Thermo Fisher Scientific), using 20 µL reaction volume.

Gene expression of 12 eQTL genes overlapping with lithium-associated DE genes were investigated using a custom designed TaqMan® Low Density Array with a total of 16 assays (Table S16), each sample run in triplicate. Reactions were run on a QuantStudio 12K Flex Real-Time PCR System (Applied Biosystems), using TaqMan™ Gene Expression Master Mix (Applied Biosystems) and 8.33 ng cDNA per well. Relative expression levels were calculated using the $\Delta\Delta Ct$ method [24], normalizing each gene of interest against the mean expression value of four endogenous control genes (Table S15). Genes with very low or no expression detected, signified by ≥80% of the samples with Ct=40, were excluded from the analysis. For each of the 12 lithium-associated genes, a DE analysis between bipolar patients and healthy

controls was performed using a simple logistic regression model in R controlling for sex and age differences. P-values were not adjusted for multiple testing.

## SUPPLEMENTARY REFERENCES


1. Bolger, A.M., M. Lohse, and B. Usadel, *Trimmomatic: a flexible trimmer for Illumina sequence data.* Bioinformatics, 2014. **30**(15): p. 2114-20.
2. Kim, D., B. Langmead, and S.L. Salzberg, *HISAT: a fast spliced aligner with low memory requirements.* Nat Methods, 2015. **12**(4): p. 357-60.
3. Liao, Y., G.K. Smyth, and W. Shi, *featureCounts: an efficient general purpose program for assigning sequence reads to genomic features.* Bioinformatics, 2014. **30**(7): p. 923-30.
4. Li, B. and C.N. Dewey, *RSEM: accurate transcript quantification from RNA-Seq data with or without a reference genome.* BMC Bioinformatics, 2011. **12**: p. 323.
5. Patro, R., et al., *Salmon provides fast and bias-aware quantification of transcript expression.* Nat Methods, 2017. **14**(4): p. 417-419.
6. Zhang, C., et al., *Evaluation and comparison of computational tools for RNA-seq isoform quantification.* BMC Genomics, 2017. **18**(1): p. 583.
7. Soneson, C., M.I. Love, and M.D. Robinson, *Differential analyses for RNA-seq: transcript-level estimates improve gene-level inferences.* F1000Res, 2015. **4**: p. 1521.
8. Newman, A.M., et al., *Robust enumeration of cell subsets from tissue expression profiles.* Nat Methods, 2015. **12**(5): p. 453-7.
9. Mancarci, B.O., et al., *Cross-Laboratory Analysis of Brain Cell Type Transcriptomes with Applications to Interpretation of Bulk Tissue Data.* eNeuro, 2017. **4**(6).
10. McKenzie, A.T., et al., *Brain Cell Type Specific Gene Expression and Co-expression Network Architectures.* Sci Rep, 2018. **8**(1): p. 8868.
11. Taylor, A.M., et al., *Axonal mRNA in uninjured and regenerating cortical mammalian axons.* J Neurosci, 2009. **29**(15): p. 4697-707.
12. Love, M.I., W. Huber, and S. Anders, *Moderated estimation of fold change and dispersion for RNA-seq data with DESeq2.* Genome Biol, 2014. **15**(12): p. 550.
13. Ulgen, E., O. Ozisik, and O.U. Sezerman, *pathfindR: An R Package for Pathway Enrichment Analysis Utilizing Active Subnetworks.* 2018: p. 272450.
14. Kanehisa, M. and S. Goto, *KEGG: kyoto encyclopedia of genes and genomes.* Nucleic Acids Res, 2000. **28**(1): p. 27-30.
15. Yu, G., et al., *clusterProfiler: an R package for comparing biological themes among gene clusters.* OMICS, 2012. **16**(5): p. 284-7.
16. The Gene Ontology, C., *The Gene Ontology Resource: 20 years and still GOing strong.* Nucleic Acids Res, 2019. **47**(D1): p. D330-D338.
17. Stahl, E.A., et al., *Genome-wide association study identifies 30 loci associated with bipolar disorder.* Nat Genet, 2019. **51**(5): p. 793-803.
18. Battle, A., et al., *Genetic effects on gene expression across human tissues.* Nature, 2017. **550**(7675): p. 204-213.
19. Ramasamy, A., et al., *Genetic variability in the regulation of gene expression in ten regions of the human brain.* Nature Neuroscience, 2014. **17**(10): p. 1418-1428.
20. Fromer, M., et al., *Gene expression elucidates functional impact of polygenic risk for schizophrenia.* Nat Neurosci, 2016. **19**(11): p. 1442-1453.
21. Ongen, H., et al., *Fast and efficient QTL mapper for thousands of molecular phenotypes.* Bioinformatics, 2016. **32**(10): p. 1479-1485.

22. Shabalin, A.A., *Matrix eQTL: ultra fast eQTL analysis via large matrix operations.* Bioinformatics, 2012. **28**(10): p. 1353-1358.
23. Benjamini, Y. and Y. Hochberg, *Controlling the False Discovery Rate - a Practical and Powerful Approach to Multiple Testing.* Journal of the Royal Statistical Society Series B-Statistical Methodology, 1995. **57**(1): p. 289-300.
24. Livak, K.J. and T.D. Schmittgen, *Analysis of relative gene expression data using real-time quantitative PCR and the 2(T)(-Delta Delta C) method.* Methods, 2001. **25**(4): p. 402-408.
25. Law, C.W., et al., *RNA-seq analysis is easy as 1-2-3 with limma, Glimma and edgeR.* F1000Res, 2016. **5**.
