# Supplementary material for: Exploring lithium’s transcriptional mechanisms of action in bipolar disorder: a multi-step study
Source: Neuropsychopharmacology. 2019 Oct 25;45(6):947–55. doi: 10.1038/s41386-019-0556-8 (PMC7162887; doi:10.1038/s41386-019-0556-8)
Supplement: Supplementary file 2 — Supplementary Figures S1 and S2 [file 41386_2019_556_MOESM2_ESM.pdf]

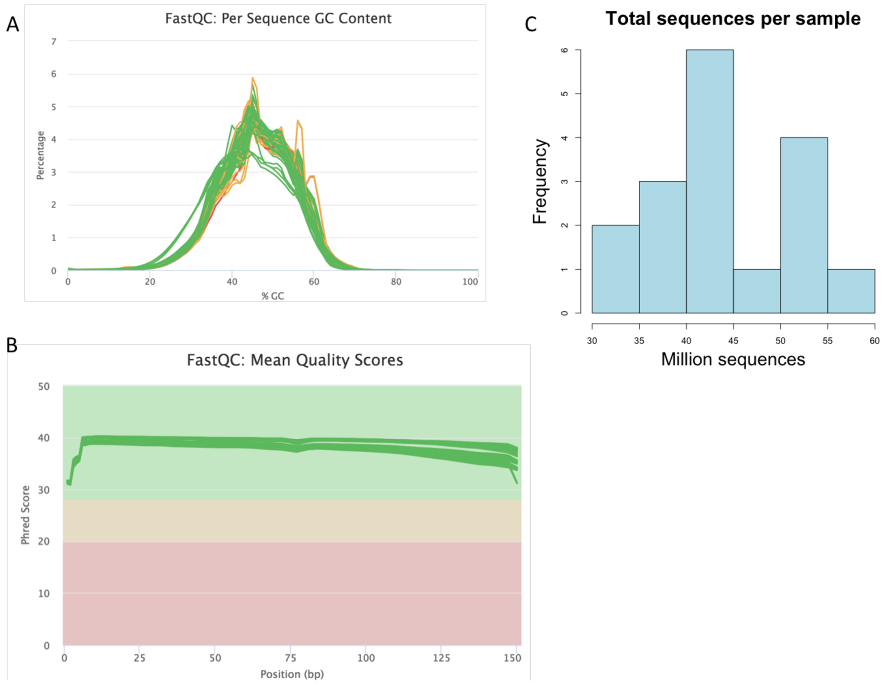


**Supplementary Figure S1. Qualitative and quantitative assessments of RNA-sequencing data. a)** Plot showing the GC content per sequence. Each line represents a sample. Most sequences had around 45% GC bases. **b)** Average quality score per base position along the sequencing read. All samples had a mean Phred score of >30 across the read, meaning that the base call accuracy was >99.9%. **c)** Total number of reads/sequences per sample. To perform a high-sensitivity RNA-sequencing analysis, we aimed for 50 million sequencing reads per sample. Due to stochastic variation during the sequencing process, reads are generally not equally distributed across samples. However, the majority of samples (12/17, 71%) received between 40-60 million reads with a mean library size of 46.5 million reads per sample.

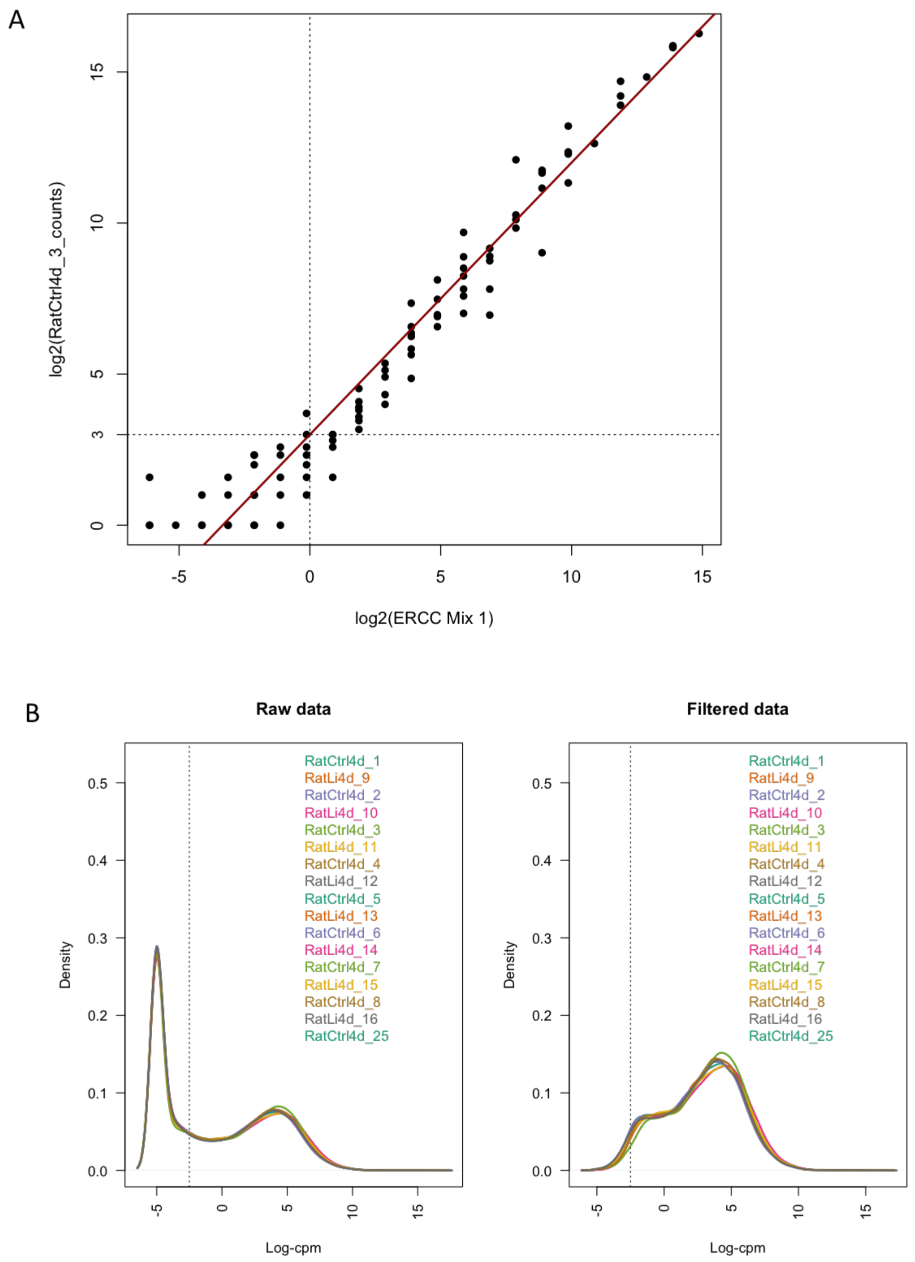


**Supplementary Figure S2. Filtering of raw sequencing data. a)** Scatter plot showing the correlation between read count estimates and the known concentrations of 92 external RNA control sequences (ERCC Mix 1) for one of the rat samples. Expression level estimates were more uncertain for ERCC sequences with the lowest concentrations as seen by the increased spread of the points below 3 read counts. Based on a visual inspection of each plot for all the samples, the filtering threshold was determined to 8 reads per gene in at least 50% of samples. **b)** Effect of pre-filtering of data. As determined by the ERCC sequence analysis, genes with less than 8 reads in more than 50% of the samples were defined as lowly or not expressed and excluded from all subsequent analyses. In total, 18148 (55.2%) low-abundance genes were filtered out. Log-CPM is the normalized read count estimate adjusted for library size. The vertical dotted lines mark the log-CPM threshold used in the filtering step. This threshold is equivalent to a CPM value of approximately 0.177, which corresponds to about 8 read counts given an average library size of 46.5 million reads. The R code used to produce this plot was adapted from [25]. CPM: Counts per million.
