# Supplementary material for: Exploring lithium’s transcriptional mechanisms of action in bipolar disorder: a multi-step study
Source: Neuropsychopharmacology. 2019 Oct 25;45(6):947–55. doi: 10.1038/s41386-019-0556-8 (PMC7162887; doi:10.1038/s41386-019-0556-8)
Supplement: Supplementary file 3 — Supplementary Figures S3 and S4 [file 41386_2019_556_MOESM3_ESM.pdf]

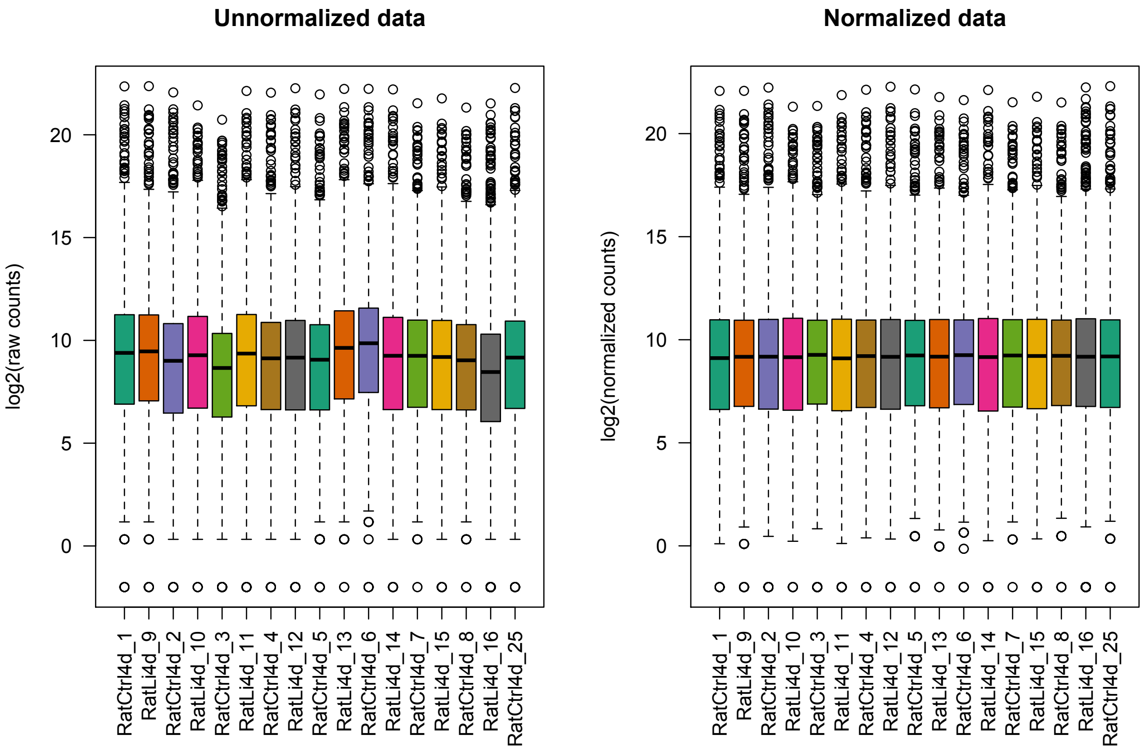

Supplementary Figure S3. Effect of normalization. The figure shows boxplots of logarithmic values for unnormalized (left) and normalized (right) expression distributions. In RNA-sequencing experiments, all samples are assumed to have a similar range and distribution of expression values. However, this assumption is often violated due to non-biological external factors, such as batch effects, that can interfere during the sample preparation or sequencing process. Normalization ensures that the expression distributions are similar across all samples, as shown in the right panel.

A

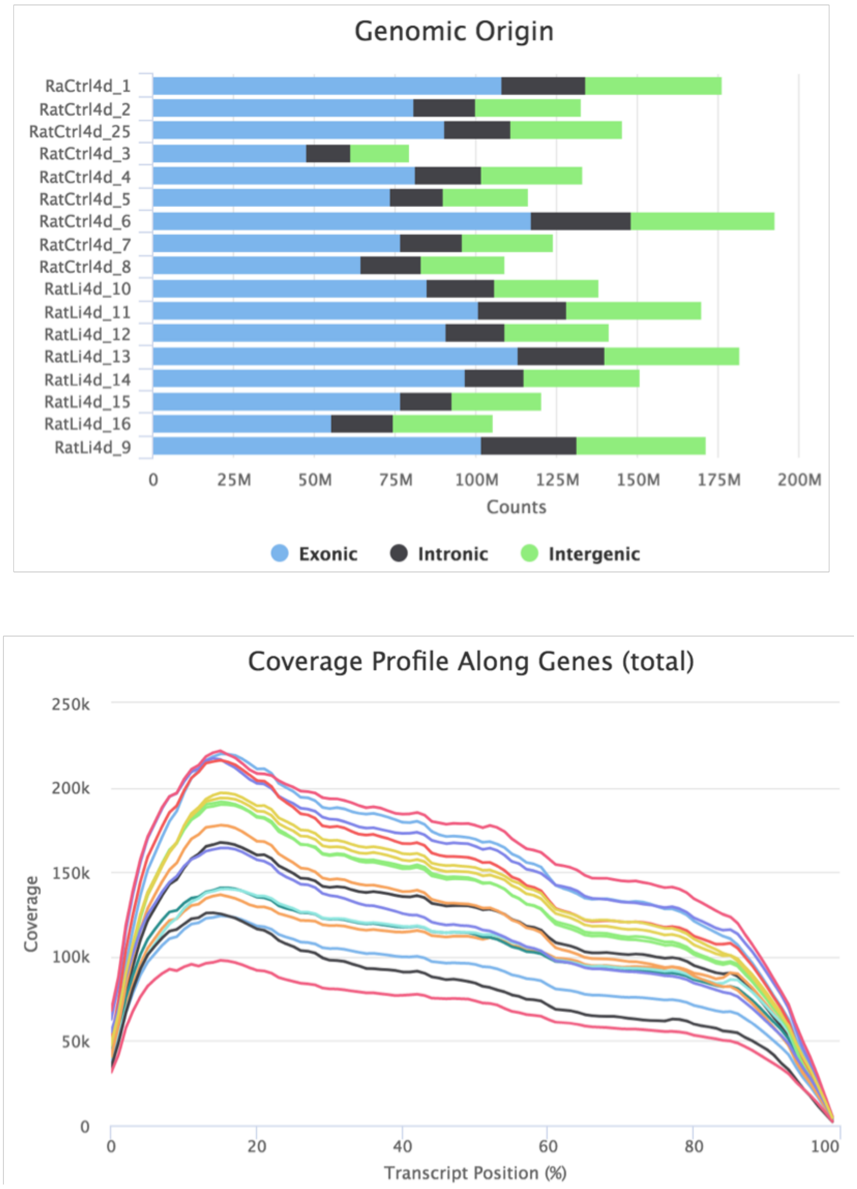

B

Supplementary Figure S4. Mapping and coverage distributions. a) The plot shows the distribution of mapped reads to either exonic, intronic, or intergenic regions of the genome. Most reads mapped to exons, but a substantial portion of reads mapped to intronic and intergenic regions. This could be due to the presence of DNA traces in the samples, novel isoforms not present in the annotation file, or because of multimapping reads, i.e. reads that map both to exonic and non-exonic regions. **b)** The plot shows the average coverage profile along the transcript for each sample. All samples had similar coverage distributions, indicating minimal transcript isoform diversity between samples. The plot also shows a tendency of 5' bias, i.e. higher coverage at the 5' end of the transcript, which is typically introduced during sample preparation. However, the has a non-specific pattern and is equal across conditions.
