# Supplementary material for: Exploring lithium’s transcriptional mechanisms of action in bipolar disorder: a multi-step study
Source: Neuropsychopharmacology. 2019 Oct 25;45(6):947–55. doi: 10.1038/s41386-019-0556-8 (PMC7162887; doi:10.1038/s41386-019-0556-8)
Supplement: Supplementary file 4 — Supplementary Figures S5 and S6 [file 41386_2019_556_MOESM4_ESM.pdf]

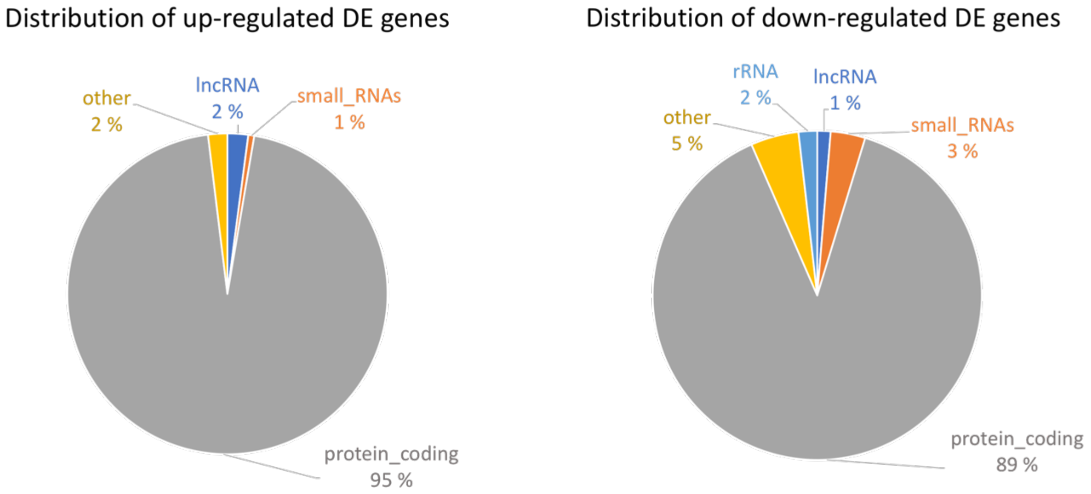


**Supplementary Figure S5. Distribution of up- and down-regulated DGE genes.** Biotypes of significantly **a)** up-regulated and **b)** down-regulated DGE genes identified in the gene-level analysis. Small RNAs include miRNAs and snoRNAs. “Other” include pseudogenes and processed pseudogenes. lncRNA: Long non-coding RNA.

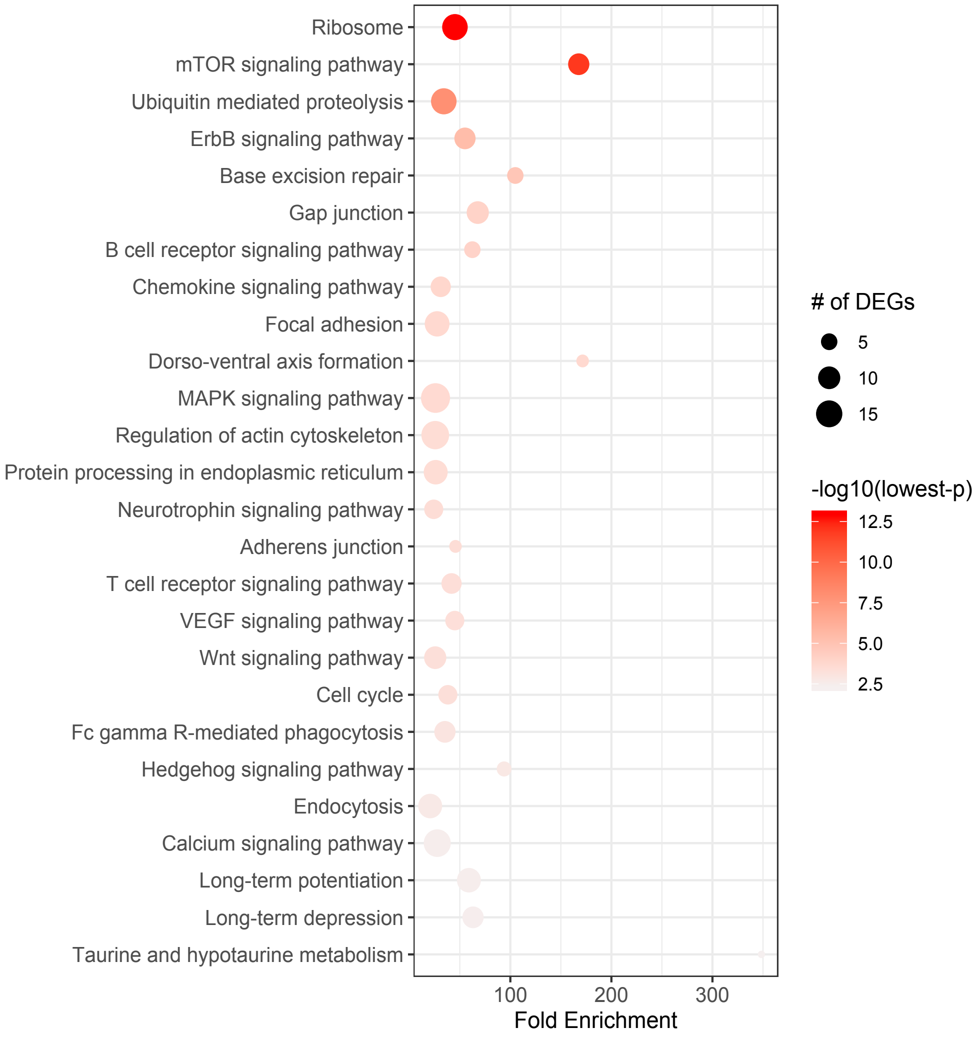


**Supplementary Figure S6. Pathway analysis of significant DGE genes.** The significant DGE genes were enriched for 26 molecular pathways. The ribosome pathway was the most significant, while MAPK signaling pathway included the largest number of DGE genes and the taurine and hypotaurine metabolism had the biggest effect size. Lowest-p refers to the lowest p-value after 10 iterations. mTOR: Mammalian target of rapamycin. MAPK: Mitogen-activated protein kinase. VEGF: Vascular endothelial growth factor.
