# Supplementary material for: Exploring lithium’s transcriptional mechanisms of action in bipolar disorder: a multi-step study
Source: Neuropsychopharmacology. 2019 Oct 25;45(6):947–55. doi: 10.1038/s41386-019-0556-8 (PMC7162887; doi:10.1038/s41386-019-0556-8)
Supplement: Supplementary file 5 — Supplementary Figures S7 and S8 [file 41386_2019_556_MOESM5_ESM.pdf]

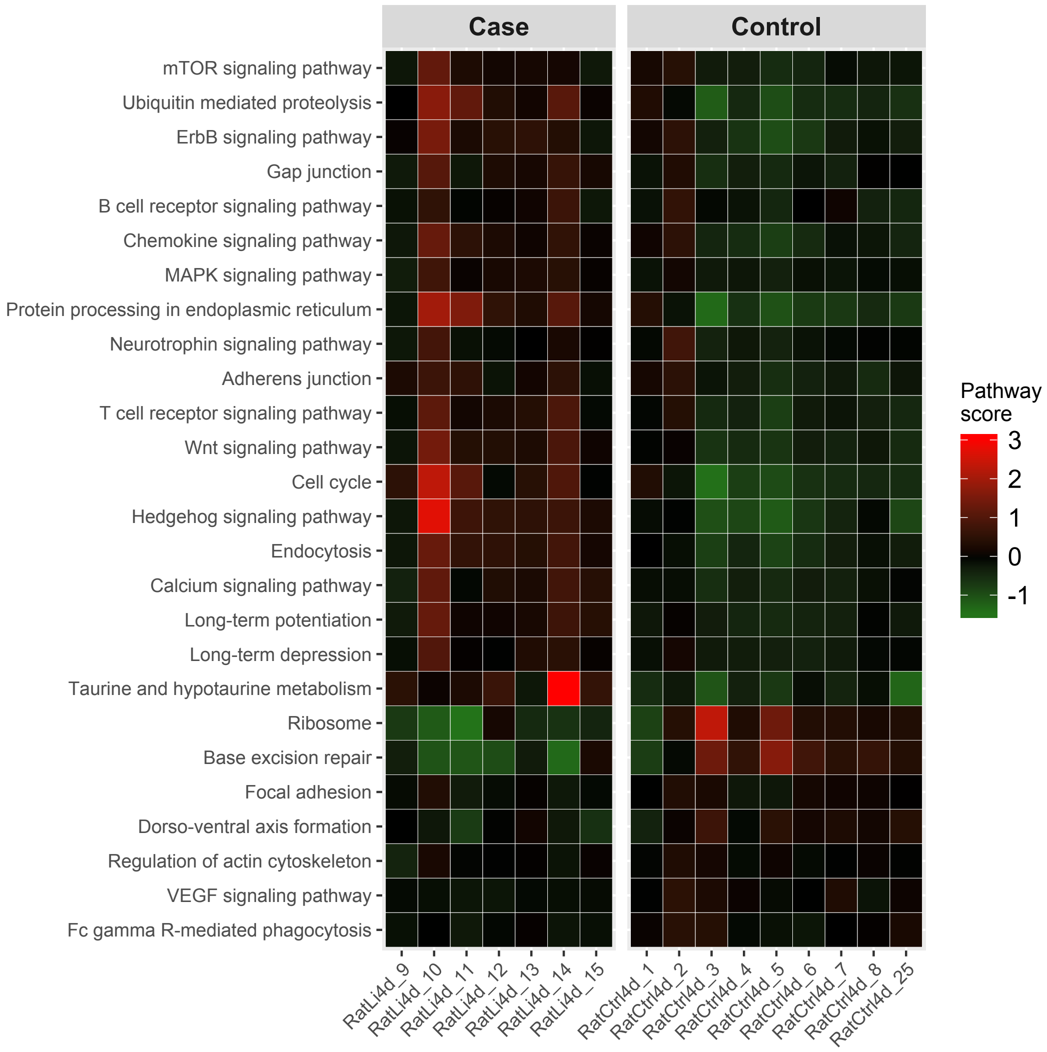

Supplementary Figure S7. Per-sample clustering of significantly enriched gene-level pathways. The pathway score is a relative measure of the extent to which a given pathway is activated (red) or repressed (green) in a given sample. It is calculated by summarizing, per sample, the standardized expression values of each gene in a pathway of interest. As the matrix shows, most enriched pathways were activated, i.e. had increased activation in lithium-treated rats (cases) compared to non-treated rats (controls).

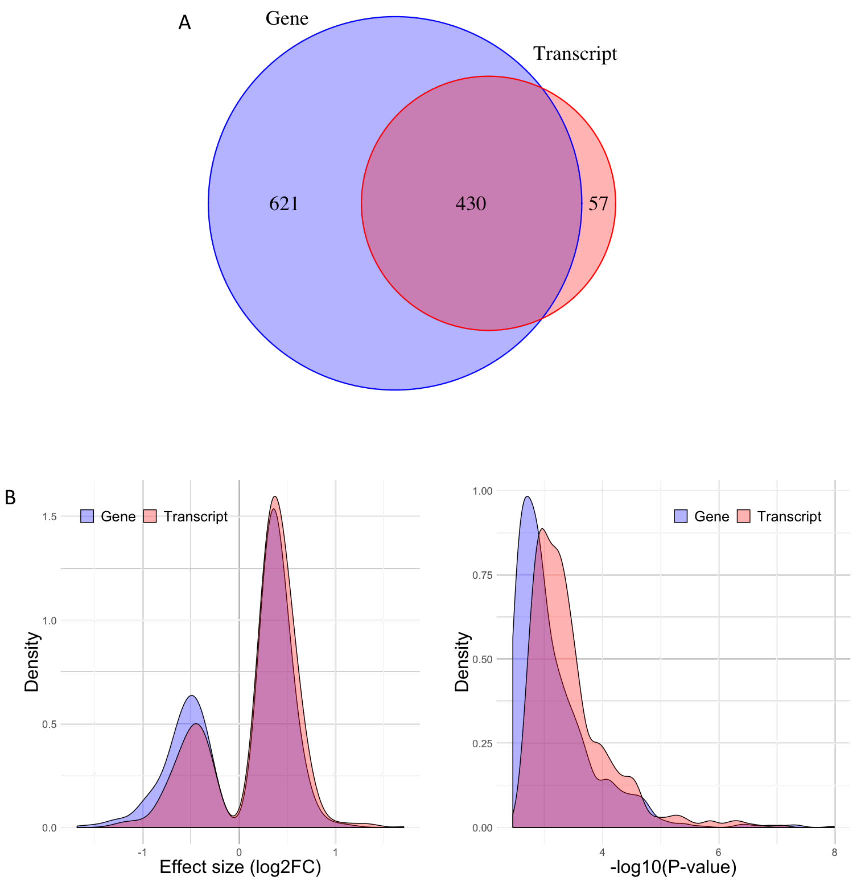

Supplementary Figure S8. Comparison between significant DGE and DTE genes. a) The transcript-level analysis identified 487 DTE genes with differential expression in lithium-treated rats. Of these, 430 (88.3%) were also identified in the gene-level analysis, while 57 (11.7%) genes were exclusively detected when individual transcripts were quantified separately. **b)** Density plots of effect sizes and p-values for the DE genes identified in the gene and transcript-level analyses, respectively. Effect sizes were highly concordant, but a slight shift towards smaller p-values is seen for the DTE genes.
