# Supplementary material for: Exploring lithium’s transcriptional mechanisms of action in bipolar disorder: a multi-step study
Source: Neuropsychopharmacology. 2019 Oct 25;45(6):947–55. doi: 10.1038/s41386-019-0556-8 (PMC7162887; doi:10.1038/s41386-019-0556-8)
Supplement: Supplementary file 6 — Supplementary Figure S9 [file 41386_2019_556_MOESM6_ESM.pdf]

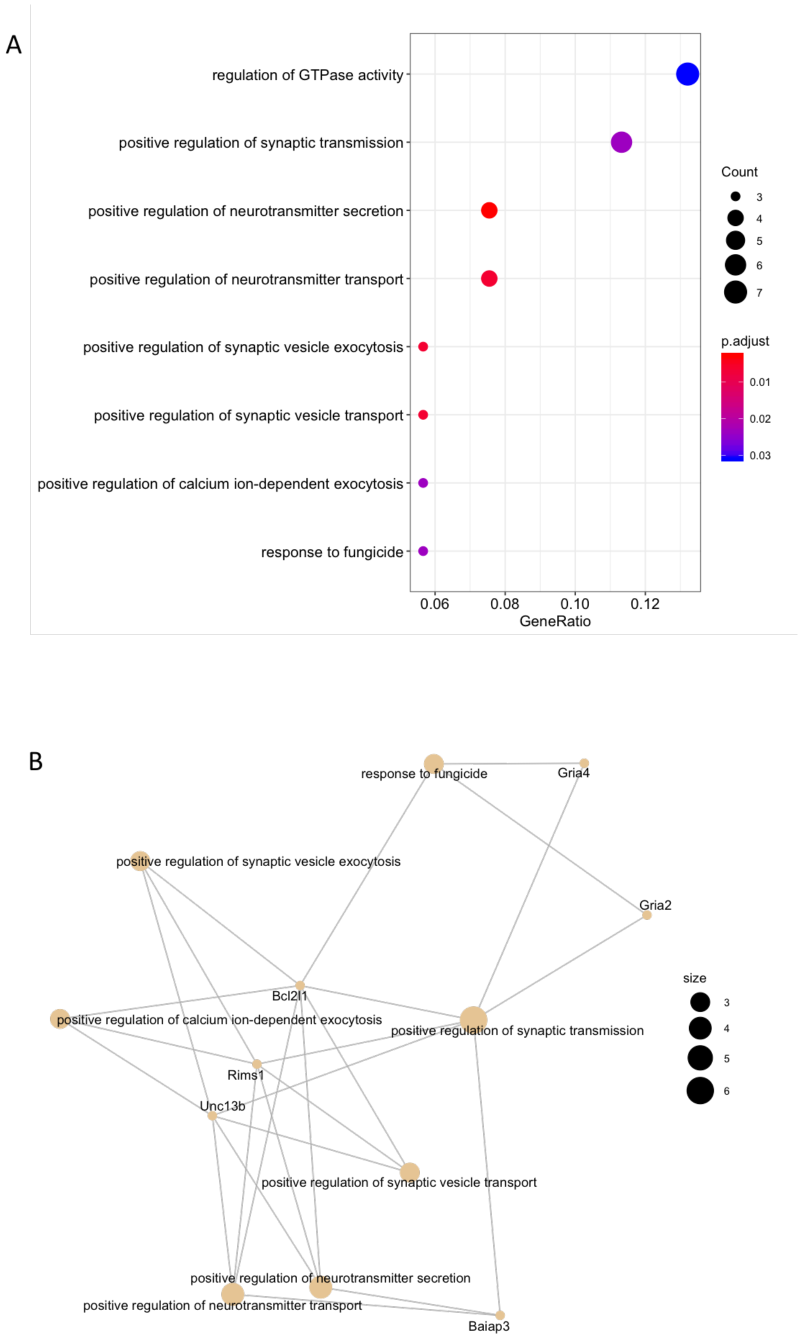

Supplementary Figure S9. GO analysis of DTE genes exclusively identified in the transcript-level analysis. a) Over-representation test of the 57 DTE-only genes resulted in 8 over-represented biological processes (GO terms). Most of these processes are involved in synaptic functions. "GeneRatio" is the number of genes related to a GO term of interest divided by the total number of genes used as input for the over-representation test. **b)** Cnet plot of over-represented GO terms forming a single cluster involving 7 interrelated GO terms (all except regulation of GTPase activity). The plot shows a high degree of overlap between the enriched GO terms, with several genes, such as *BCL2L1* and *RIMS1*, being involved in five or more biological processes.
